# Tranexamic acid and bleeding in patients treated with non-vitamin K oral anticoagulants undergoing dental extraction: The EXTRACT-NOAC randomized clinical trial

**DOI:** 10.1371/journal.pmed.1003601

**Published:** 2021-05-03

**Authors:** Anna Ockerman, Isabel Miclotte, Maarten Vanhaverbeke, Thomas Vanassche, Ann Belmans, Jan Vanhove, Joeri Meyns, Nasser Nadjmi, Geert Van Hemelen, Patrick Winderickx, Reinhilde Jacobs, Constantinus Politis, Peter Verhamme

**Affiliations:** 1 Oral and Maxillofacial Surgery—Imaging and Pathology Research Group, Department of Imaging and Pathology, University of Leuven and Department of Oral & Maxillofacial Surgery, University Hospitals Leuven, Leuven, Belgium; 2 Department of Cardiovascular Medicine, University Hospitals Leuven, Leuven, Belgium; 3 Leuven Biostatistics and Statistical Bioinformatics Centre, Department of Public Health and Primary Care, University of Leuven, Leuven, Belgium; 4 Oral & Maxillofacial Surgery, Regional Hospital Heilig Hart Leuven, Leuven, Belgium; 5 Oral & Maxillofacial Surgery, General Hospital St-Jan Genk, Genk, Belgium; 6 Department of Cranio-Maxillofacial Surgery, University of Antwerp, Antwerp, Belgium; 7 Oral & Maxillofacial Surgery, ZMACK Association, AZ Monica Antwerp, Antwerp, Belgium; 8 Department of Dental Medicine, Karolinska Institutet, Stockholm, Sweden

## Abstract

**Background:**

Oral bleeding after dental extraction in patients on non-vitamin K oral anticoagulants (NOACs) is a frequent problem. We investigated whether 10% tranexamic acid (TXA) mouthwash decreases post-extraction bleeding in patients treated with NOACs.

**Methods and findings:**

The EXTRACT-NOAC study is a randomized, double-blind, placebo-controlled, multicenter, clinical trial. Patients were randomly assigned to 10% TXA or placebo mouthwash and were instructed to use the mouthwash once prior to dental extraction, and thereafter for 3 times a day for 3 days. The primary outcome was the number of patients with any post-extraction oral bleeding up to day 7. Secondary outcomes included periprocedural, early, and delayed bleeding, and the safety outcomes included all thrombotic events. The first patient was randomized on February 9, 2018 and the last patient on March 12, 2020. Of 222 randomized patients, 218 patients were included in the full analysis set, of which 106 patients were assigned to TXA (74.8 (±8.8) years; 81 men) and 112 to placebo (72.7 (±10.7) years; 64 men). Post-extraction bleeding occurred in 28 (26.4%) patients in the TXA group and in 32 (28.6%) patients in the placebo group (relative risk, 0.92; 95% confidence interval [CI], 0.60 to 1.42; *P* = 0.72). There were 46 bleeds in the TXA group and 85 bleeds in the placebo group (rate ratio, 0.57; 95% CI, 0.31 to 1.05; *P* = 0.07). TXA did not reduce the rate of periprocedural bleeding (bleeding score 4 ± 1.78 versus 4 ± 1.82, *P* = 0.80) and early bleeding (rate ratio, 0.76; 95% CI, 0.42 to 1.37). Delayed bleeding (rate ratio, 0.32; 95% CI, 0.12 to 0.89) and bleeding after multiple extractions (rate ratio, 0.40; 95% CI, 0.20 to 0.78) were lower in the TXA group. One patient in the placebo group had a transient ischemic attack while interrupting the NOAC therapy in preparation for the dental extraction. Two of the study limitations were the premature interruption of the trial following a futility analysis and the assessment of the patients’ compliance that was based on self-reported information during follow-up.

**Conclusions:**

In patients on NOACs undergoing dental extraction, TXA does not seem to reduce the rate of periprocedural or early postoperative oral bleeding compared to placebo. TXA appears to reduce delayed bleeds and postoperative oral bleeding if multiple teeth are extracted.

**Trial registration:**

ClinicalTrials.gov NCT03413891

EudraCT; EudraCT number:2017-001426-17; EudraCT Public website: eudract.ema.europa.eu.

## Abstract 2

Anna Ockerman and co-workers evaluate mouthwash containing tranexamic acid for people on non-vitamin K oral anticoagulants undergoing dental extraction.

## Author summary

**Why was this study done?:**

- Oral bleeding after dental extraction in patients on non-vitamin K oral anticoagulants (NOACs) is a frequent problem and may occur in up to 25% of the patients.
- Because of bleeding, patients often reconsult a dentist or surgeon and might need a reintervention to stop the bleeding. Patients might also interrupt their anticoagulant treatment, which could lead to subsequent life-threatening thrombotic events.
- There is no consensus about the best hemostatic management to reduce bleeding after dental extraction in these patients.
- Tranexamic acid (TXA) was proven to reduce bleeding in other surgical settings and is a cheap and widely available hemostatic agent.

**What did the researchers do and find?:**

- We performed a randomized, double-blind, placebo-controlled, multicenter, clinical trial to evaluate if the use of a TXA mouthwash reduces bleeding.
- TXA did not seem to affect the rate of periprocedural or early postoperative oral bleeding compared to placebo.
- TXA appeared to reduce delayed bleeding and postoperative bleeding if multiple teeth were extracted.
- The use of TXA might also reduce the number of patients reconsulting a dentist or surgeon after dental extraction.

**What do these findings mean?:**

- Our results confirm the high event rate of oral bleeds in patients treated with NOACs undergoing dental extraction, with more than 1 out of 4 patients reporting any bleeding.
- TXA might help to reduce delayed post-extraction bleeding and bleeding after multiple dental extractions in patients on NOACs and lead to less patients reconsulting healthcare services.

## Introduction

Oral bleeding is a frequent complication after dental extraction in anticoagulated patients and might occur in up to 25% of these patients [1,2]. A meta-analysis on the risk of post-extraction bleeding pointed out that these patients have a 3 times higher risk of bleeding than patients not taking anticoagulant drugs [3]. Bleeding often results in patients reconsulting a dentist or oral and maxillofacial surgeon and may require a reintervention. These unplanned visits to the dental practice or hospital increase healthcare costs. In addition, if the anticoagulant therapy is interrupted, bleeding may turn into a risk factor for thrombotic events.

Although the number of patients treated with direct or non-vitamin K oral anticoagulants (DOACs or NOACs) is increasing, data on how to prevent and manage bleeding in patients undergoing dental extractions are limited [4]. Moreover, evidence from clinical trials shows a potential increased risk of mucosal bleeding in certain NOAC regimens [5]. Guidelines advise performing dental extraction at trough level of a NOAC, which can be implemented by skipping a NOAC dose on the morning of the day of the dental extraction [6]. This strategy appeared to be safe in a recent prospective pilot study, although there was a signal toward excess delayed bleeding compared to non-anticoagulated patients [1]. Based on these preliminary findings, the current clinical trial was designed.

Tranexamic acid (TXA) is an attractive hemostatic agent for oral surgery as it can be applied locally, resulting in low systemic absorption [7,8]. Previous research has shown that the use of TXA mouthwash is effective in decreasing bleeding after dental extraction in patients treated with vitamin K antagonists, but its use in NOAC-treated patients has not yet been studied [9,10].

The current interventional EXTRACT-NOAC study was designed to assess whether a 10% TXA mouthwash reduces bleeding after dental extraction in patients on NOACs.

## Methods

The EXTRACT-NOAC study is a prospective, randomized, double-blind, placebo-controlled, multicenter, investigator-initiated, clinical trial. The study obtained written consent by the Medical Ethics Committee of University Hospitals Leuven in July 2017 (S60131). The trial is registered at ClinicalTrials.gov (Identifier: NCT03413891). The full and detailed rationale and design of the study have been extensively described previously [11]. This study is reported following the Consolidated Standards of Reporting Trials (CONSORT) guidelines (S1 CONSORT Checklist).

### Patients

Patients older than 18 years were eligible if they were treated with a NOAC (rivaroxaban, apixaban, edoxaban, or dabigatran), were scheduled for a dental extraction, and provided written informed consent. Patients who were pregnant or lactating were excluded [11]. Patients visiting the oral and maxillofacial departments of the following Belgian hospitals were screened for eligibility: University Hospitals Leuven, Regional Hospital Heilig Hart Leuven, General Hospital St-Jan Genk, and AZ Monica Antwerp.

### Randomization and study procedures

All patients were instructed to skip the morning dose of their NOAC on the day of the dental extraction, in agreement with the European Hearth Rhythm Association (EHRA) guidelines [6] and as previously validated by our group [1]. No other precoagulation screening was performed, in agreement with the EHRA guidelines [6]. If the procedure was planned early in the day, it was also allowed to skip the anticoagulant dose the evening before, aiming for an 18 to 24 hours window before the last dose. Patients were randomized through an interactive web recognition system to a 1g/10 mL (10%) TXA mouthwash or a taste and color-matching placebo, both manufactured by the Leuven Centre for Clinical Pharmacology, University Hospitals Leuven, Belgium. A computer-generated block randomization list was generated by an independent person (Leuven Clinical Coordinating Centre, University of Leuven, Belgium) for treatment allocation. The randomization was not stratified. Patients were enrolled in the study and assigned to the study interventions by clinicians. Patients, clinicians, and treating surgeons were blinded to the allocated intervention.

Patients were instructed to use the mouthwash immediately before the dental extraction and subsequently 3 times a day for 3 days starting the day after the extraction. They were asked to use the mouthwash for 1 minute and to spit it out afterwards. Extraction wound management was left at the discretion of the physician. Patients were instructed to resume the NOAC the day after dental extraction unless there were hemostasis issues. Investigators blinded to the allocated treatment contacted the patients by phone on day 2 and day 7 after dental extraction to assess compliance with the study protocol, the occurrence of any bleeding, and other secondary and safety outcomes [11].

### Outcomes

The primary outcome was the number of patients with any oral bleeding, defined as overt bleeding within the oral cavity. Secondary outcomes were the number of bleeding events in the different bleeding categories, periprocedural bleeding (defined as a bleeding score measured on a visual analogue scale ranging from 0 to 10), unplanned medical contacts, reinterventions following oral bleeding, and unplanned NOAC interruptions. Oral bleeding events were categorized as minor, clinically relevant, or major, as previously published [1], and as early (on the day of the extraction or the day after) or delayed (on day 2 or later) [11]. Facial hematomas were recorded as well. A blinded adjudicator adjudicated all suspected bleeding events and reinterventions. Safety outcomes were thrombotic events, including myocardial infarction, stroke, systemic embolism, and venous thromboembolism, any allergic reactions to the mouthwash, and non-oral bleeding events (such as epistaxis, eye bleeding, or bruises), up to the end of the study follow-up [11].

### Statistical analysis

The study was designed to test the hypothesis that TXA mouthwash would be superior to placebo with respect to the number of patients with any oral bleeding. Based on a pilot study, the sample size was set at 236 patients, which would allow to detect a treatment difference of 15% between the TXA and placebo group using a chi-squared test with an expected proportion of patients with any bleeding of 30% in the control group [1]. The statistical power was set at 80% and the 2-sided significance level at 5%. All analyses were performed using SAS version 9.4 and SAS/STAT version 15.1 for Windows.

The primary analysis was conducted in the full analysis set, which included all randomized patients, but excluded 3 patients for whom no follow-up data could be obtained and 1 control patient who used off-trial TXA [12]. A sensitivity analysis was performed whereby the primary endpoint for these patients was imputed according to a worst-case scenario (i.e., occurrence of oral bleeding), but yielded similar results. A further sensitivity analysis was performed on the per-protocol set, additionally excluding patients who had not complied with the assigned treatment for at least 80%.

The primary outcome (i.e., number of patients with any oral bleeding) was analyzed using a chi-squared test. The effect of treatment was estimated by the risk ratio and presented along with its 95% confidence interval (CI).

The secondary outcomes were analyzed, and a prespecified subgroup analysis was performed, by means of a logistic regression (for the number of patients with bleeds) and negative binomial regression model (for the number of bleeds). The CIs from the negative binomial regression were calculated using the normal approximation. The treatment effect was estimated as a rate ratio (the number of bleeds per patient during the first 7 days after dental extraction). The subgroup analysis included a risk analysis for bleeding events accounting for patient demographics and procedural characteristics, stratified per treatment group. Additionally, a post hoc exploratory analysis was performed, analyzing all oral bleeds plus facial hematomas by means of a logistic regression (for the primary endpoint) and negative binomial regression model (for the number of bleeds).

Importantly, an independent Data Safety Monitoring Board performed an unplanned interim analysis on the number of patients with oral bleeding in May 2020 with the aim of assessing futility or reassessing the sample size. At that time, the trial was suspended because of the outbreak of the Severe Acute Respiratory Syndrome Coronavirus 2 (SARS-CoV-2), and 222 of the 236 planned patients had been randomized. The Data Safety Monitoring Board recommended not to reinitiate the study based on the number of patients with bleeding. The study team remained blinded to all study results. The full details of the methodology of the interim and full analysis are outlined in S1 SAP Interim and S2 SAP Final, respectively.

## Results

### Recruitment, baseline characteristics, and follow-up

**Fig 1:**
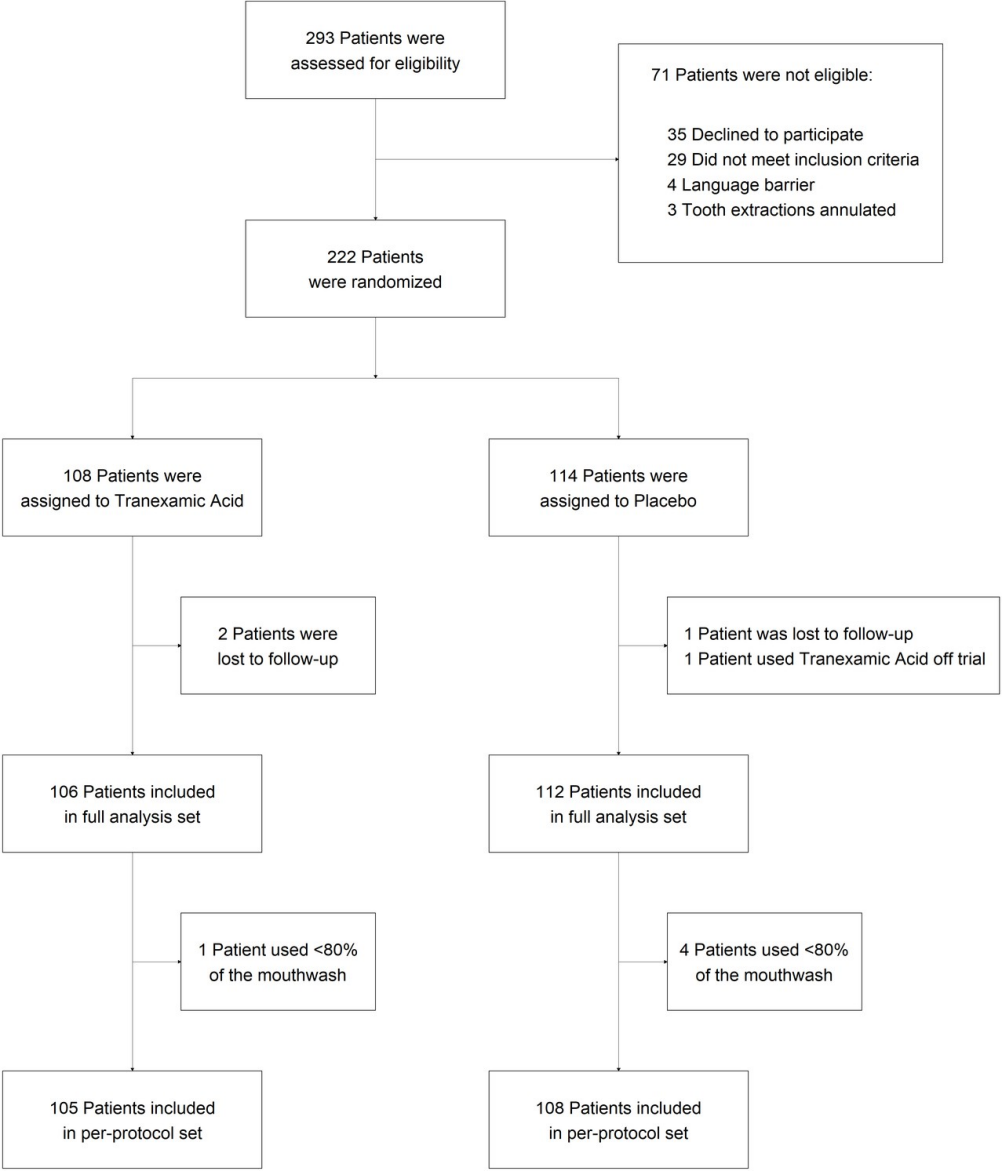
Screening, randomization, follow-up, and analysis of patients.

The study flowchart is shown in Fig 1. The first patient was enrolled in February 2018, and the trial was closed in May 2020. Of 293 patients referred for dental extraction, 222 eligible patients were randomized: 108 patients were assigned to TXA mouthwash and 114 to placebo. Three patients were lost to follow-up, and 1 patient took off-trial TXA, so that 106 TXA-treated patients and 112 placebo-treated patients were included in the full analysis set.

The baseline characteristics of the patients are shown in Table 1. The 2 groups were well balanced, except for a difference in sex. The mean age was 74.8 (±8.8) years in the TXA group and 72.7 (±10.7) years in the placebo group. Details of the dental extractions are presented in S1 Table.

**Table 1:** Baseline characteristics of the patients.

Characteristics	TXA (*N* = 106)	Placebo (*N* = 112)
Age–year*	74.8 ± 8.8	72.7 ± 10.7
Male sex–no. (%)	81 (76.4)	64 (57.1)
Smoking status–no. (%)	-	-
Active smokers	10 (9.4)	15 (13.4)
History of smoking	53 (50.0)	47 (42.0)
Never smoked	43 (40.6)	50 (44.6)
Alcohol consumption–no. (%)		
≤2 units daily	99 (93.4)	105 (93.8)
>2 units daily	7 (6.6)	7 (6.3)
Medical background–no. (%)^†^	-	-
Chronic heart failure	29 (27.4)	24 (21.4)
Hypertension	76 (71.7)	76 (67.9)
Diabetes	21 (19.8)	15 (13.4)
Stroke	21 (19.8)	29 (25.9)
Coronary artery disease	35 (33.0)	36 (32.1)
Peripheral artery disease	11 (10.4)	9 (8.0)
CHADS-VASc score	3.7 ± 1.7	3.7 ± 1.6
NOAC type–no. (%)	-	-
Rivaroxaban	38 (35.8)	40 (35.7)
Apixaban	29 (27.4)	33 (29.5)
Edoxaban	21 (19.8)	19 (17.0)
Dabigatran	18 (17.0)	20 (17.9)
Indication for NOAC–no. (%)	-	-
Atrial fibrillation	88 (83.0)	88 (78.6)
Venous thromboembolism	11 (10.4)	14 (12.5)
Other or unknown	7 (6.6)	10 (8.9)

Percentages may not total 100 because of rounding.

* Plus-minus values are means ± SD.

† These data are not mutually exclusive.

NOAC, non-vitamin K oral anticoagulant; TXA, tranexamic acid.

### Primary and secondary bleeding outcomes

The primary and secondary bleeding outcomes are summarized in Table 2. There was no difference in the number of patients with any oral post-extraction bleeding in the TXA group (28 patients, 26.4%) versus the placebo group (32 patients, 28.6%) (relative risk, 0.92; 95% CI, 0.60 to 1.42; *P* = 0.72). When assessing all oral bleeding events, there were 46 oral bleeds in the TXA group versus 85 in the placebo group (rate ratio, 0.57; 95% CI, 0.31 to 1.04, *P* = 0.07). The procedural bleeding score was similar between both groups: 4 ± 1.78 on a VAS scale (0 to 10) for TXA-treated patients and 4 ± 1.82 for placebo-treated patients (*P* = 0.80).

**Table 2:** Primary and secondary bleeding outcomes.

Outcome	Patients with bleeding–no. (%)			Number of bleeds–no. (%)		
	TXA	Placebo	Relative risk	TXA	Placebo	Rate ratio
	(*N* = 106)	(*N* = 112)	(95% CI)	(*N* = 106)	(*N* = 112)	(95% CI)
**Primary outcome**	-	-	-	-	-	-
Any oral bleeding	**28 (26.4)**	**32 (28.6)**	**0.93 (0.60; 1.42)**	**-**	**-**	**-**
**Secondary outcomes**	-	-	-	-	-	-
Oral bleeding*	-	-	-	46	85	0.57 (0.31; 1.05)
Clinically relevant	4 (3.8)	10 (8.9)	0.42 (0.14; 1.31)	5	13	0.44 (0.14; 1.42)
Minor	27 (25.5)	29 (25.9)	0.98 (0.63; 1.55)	41	72	0.60 (0.32; 1.12)
Delayed	7 (6.6)	17 (15.2)	0.44 (0.19; 1.01)	11	36	0.32 (0.12; 0.89)
Early	25 (23.6)	27 (24.1)	0.98 (0.61; 1.57)	35	49	0.76 (0.42; 1.37)
Facial hematomas*	6 (5.7)	14 (12.5)	0.45 (0.18; 1.14)	6	14	0.45 (0.17; 1.18)
**Total bleeding**	**31 (29.2)**	**40 (35.7)**	**0.82 (0.56; 1.21)**	**52**	**99**	**0.56 (0.32; 0.96)**

The rate ratios are obtained by means of a negative binomial regression model.

* Patients may have had both clinically relevant and minor oral bleeds, may have had both delayed and early oral bleeds, and may have had oral bleeding plus a facial hematoma or only a facial hematoma.

CI, confidence interval; TXA, tranexamic acid.

Patients in the TXA group had less delayed oral bleeds than patients in the placebo group (11 versus 36 delayed bleeds, rate ratio, 0.32; 95% CI, 0.12 to 0.89). TXA-treated patients had fewer bleeds related to the dental extraction, which were oral bleeds and facial hematomas, compared to patients assigned to placebo (52 versus 99 bleeds, rate ratio, 0.56; 95% CI, 0.32 to 0.96).

All oral bleeds in this trial were managed with local hemostatic measures, except for one major oral bleeding in a patient in the placebo arm who needed a blood transfusion and who was hospitalized for 2 days.

**Fig 2:**
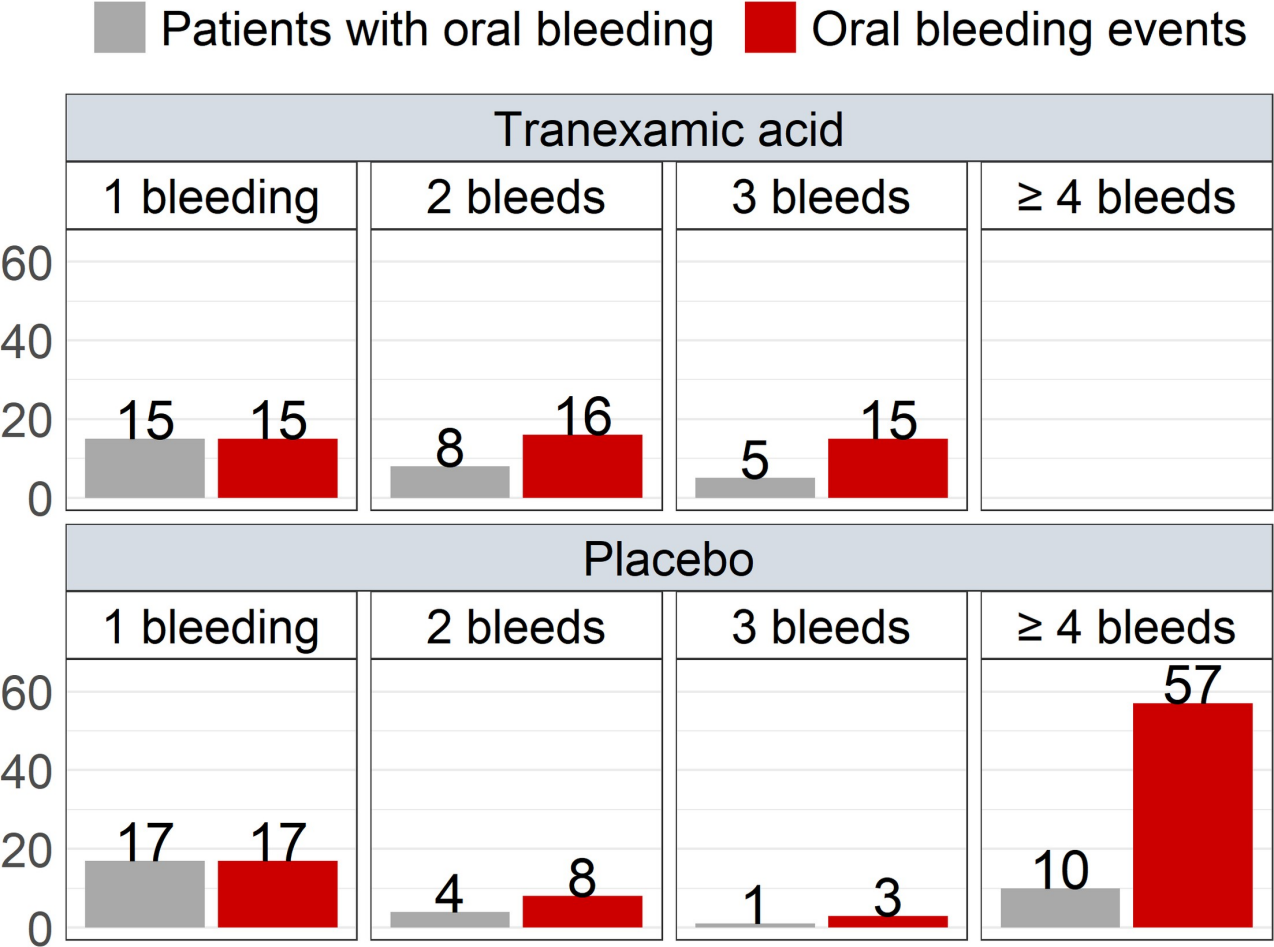
Post-extraction oral bleeds in patients who suffered from 1, 2, 3, or ≥4 bleeds.

**Fig 3:**
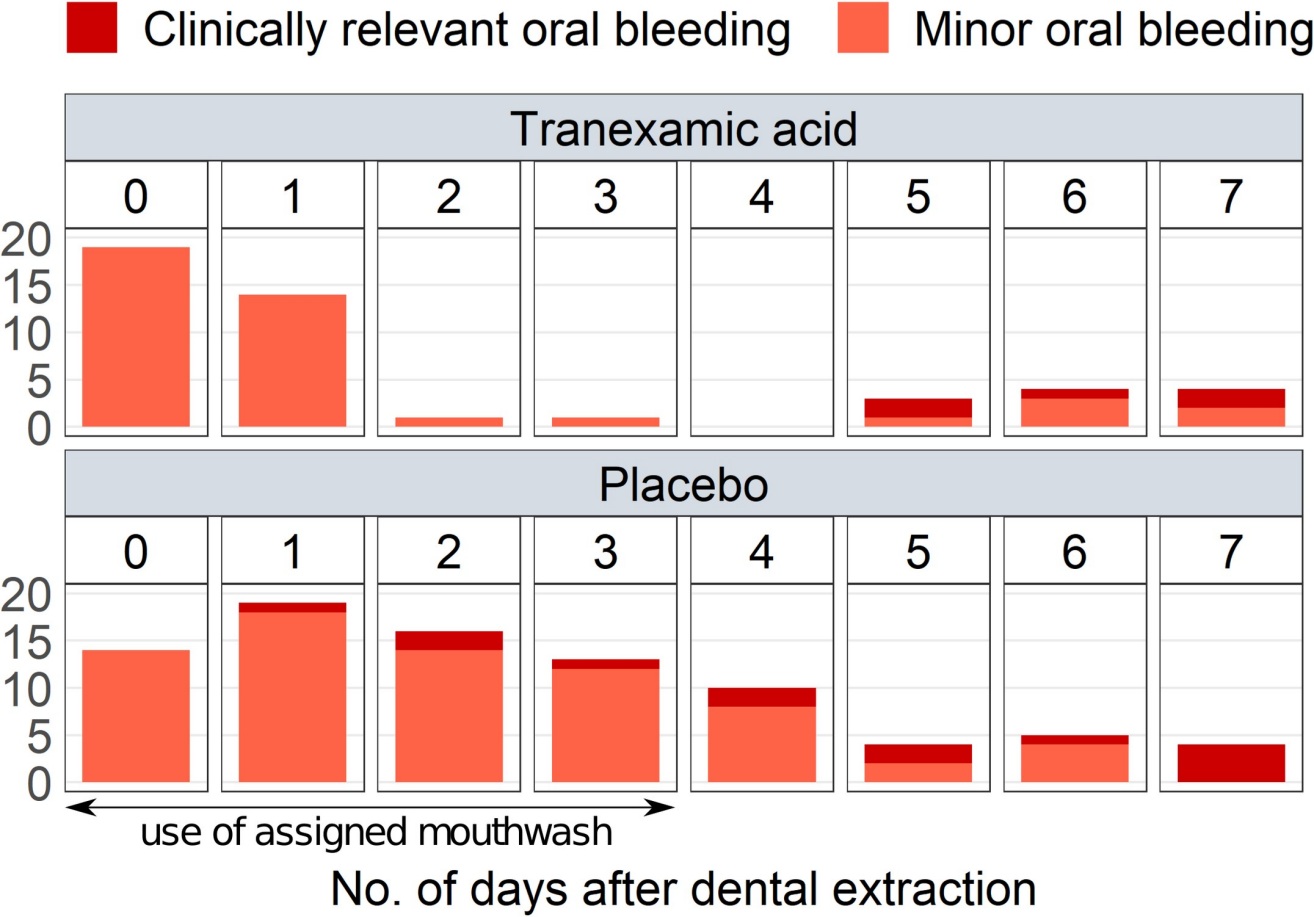
Clinically relevant and minor oral bleeds by day after dental extraction.

Of the 60 patients who reported oral bleeding, 28 experienced more than 1 bleeding (Fig 2). No patient had 4 or more oral bleeds in the TXA group, whereas 10 patients in the placebo group had. Fig 3 shows the number of oral bleeds per day after dental extraction. TXA had no influence on early post-extraction oral bleeding (day 0 and day 1) (see also Table 2). In contrast, the number of oral bleeds was lower on days 2, 3, and 4 after extraction for patients in the TXA group compared to the placebo group (see also Table 2).

### Other secondary outcomes

Fewer patients treated with TXA (6.6%) had an unplanned medical contact after dental extraction compared to patients using placebo (16.1%) (relative risk, 0.41; 95% CI, 0.18 to 0.94) (Table 3). The most frequent reasons for unplanned medical contact were bleeding and infection (S2 Table). The number of patients requiring a reintervention for oral bleeding or that interrupted their NOAC was comparable between both groups (Table 3). The majority of unplanned NOAC interruptions was because of bleeding complications (S2 Table).

**Table 3:** Other secondary and safety outcomes.

Outcome	Number of patients–no. (%)			Number of events–no. (%)		
	TXA	Placebo	Relative risk	TXA	Placebo	Rate ratio
	(*N* = 106)	(*N* = 112)	(95% CI)	(*N* = 106)	(*N* = 112)	(95% CI)
**Secondary outcomes**	-	-	-	-	-	-
Unplanned medical contact	7 (6.6)	18 (16.1)	0.41 (0.18; 0.94)	10	27	0.39 (0.16; 0.98)
Reintervention after oral bleeding	4 (3.8)	10 (8.9)	0.42 (0.13; 1.31)	7	13	0.57 (0.17; 1.91)
Unplanned NOAC interruption	6 (5.7)	9 (8.0)	0.70 (0.26; 1.91)	6	9	-
**Safety outcomes**	-	-	-	-	-	-
Thrombotic events*	0	1 (0.9)	-	0	1	-
Allergic reactions	2 (1.9)	2 (1.8)	1.06 (0.15; 7.37)	2	2	-
Epistaxis	2 (1.9)	4 (3.6)	0.53 (0.10; 2.83)	2	9	-
Eye bleeding	1 (0.9)	2 (1.8)	0.53 (0.05; 5.74)	1	2	-
Bruises^†^	2 (1.9)	4 (3.6)	0.53 (0.10; 2.83)	2	4	-
Hematuria or blood in faces	1 (0.9)	1 (0.9)	0.95 (0.06; 15.3)	1	1	-

The rate ratios are obtained by means of a negative binomial regression model.

* One patient suffered from a transient ischemic attack while interrupting the NOAC in preparation for the tooth extraction.

† Bruising caused by, for example, bumping a limb or body part into something, or after blood sampling.

NOAC, non-vitamin K oral anticoagulant; TXA, tranexamic acid.

### Safety outcomes

Only 1 patient included in the placebo group suffered from a thrombotic event in preparation for dental extraction (Table 3). The participant had stopped NOAC treatment 2 days before dental extraction and had a transient ischemic attack. There were no thrombotic events in the TXA group. Allergic reactions were rare. Two patients in both treatment groups reported a tingling feeling in their mouth after using the mouthwash (Table 3).

### Subgroup analysis

**Fig 4:**
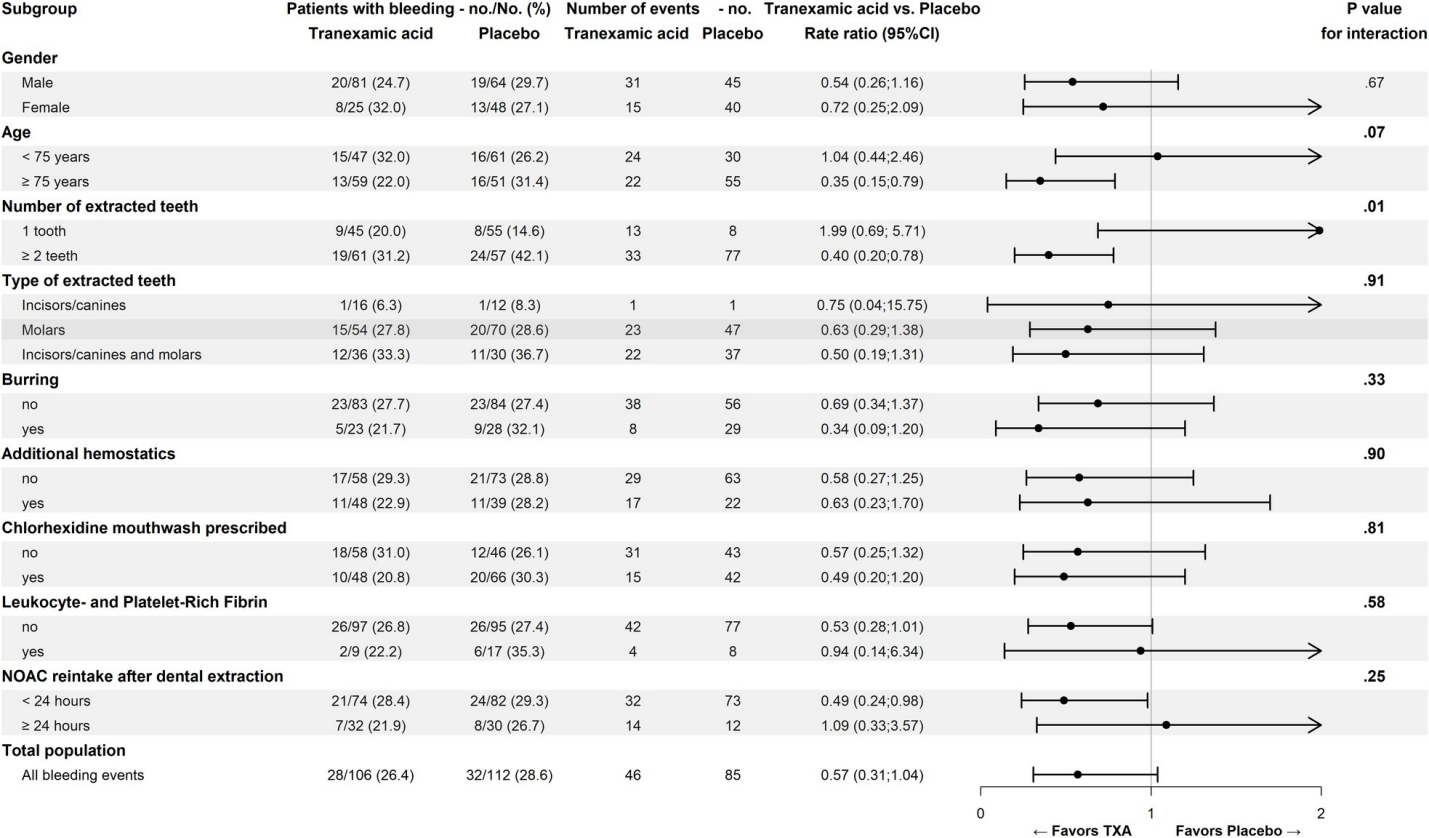
Estimated rate of oral bleeding according to patient and surgical characteristics. no./No. = the number of patients with oral bleeds divided by the total number of patients in the subgroup; no. = number of oral bleeding events. The rate ratios are given for the number of oral bleeding events and are obtained by means of a negative binomial regression. CI, confidence interval; NOAC, non-vitamin K oral anticoagulant; TXA, tranexamic acid.

The exploratory subgroup analysis is shown in Fig 4. A significant treatment interaction was identified in patients requiring extraction of 2 or more teeth. In this subgroup, there were 33 bleeds in 19/61 (31.2%) patients assigned to TXA and 77 bleeds in 24/57 (42.2%) of patients assigned to placebo (rate ratio, 0.40; 95% CI, 0.20 to 0.78). In patients who were 75 years or older, there were 24 bleeds in 13/59 (22.0%) patients assigned to TXA and 55 bleeds in 16/51 (31.4%) patients assigned to placebo (rate ratio, 0.35; 95% CI, 0.15 to 0.79), although this interaction did not reach statistical significance (*P* = 0.07).

## Discussion

To our knowledge, this is the first randomized double-blind clinical trial assessing the efficacy of TXA to reduce post-extraction bleeding in patients treated with NOACs. The results of the current trial provide new clinical data in the debate of the optimal anticoagulant and hemostatic management of patients on NOACs undergoing dental extractions [4]. We observed no reduction in the number of patients with oral bleeding when comparing TXA mouthwash with placebo. TXA did not reduce the rate of periprocedural bleeding or early bleeding after extraction, but reduced delayed bleeding and bleeding after multiple dental extractions. Patients using TXA also had fewer unscheduled medical contacts after dental extraction (59% reduction), predominantly related to bleeding.

Our results confirm the high event rate of oral bleeds in patients treated with NOACs undergoing tooth extraction, with more than 1 out of 4 patients reporting any bleeding. Moreover, half of the patients with a bleeding reported multiple oral bleeding events. In the TXA group, no patients had 4 or more bleedings, compared to 10 patients in the placebo group. Fewer patients in the TXA group thus suffered recurrent bleeding.

Two patients in each group experienced allergic reactions after the use of the assigned mouthwash, and no patients had thrombotic complications after the use of TXA, highlighting that TXA is well tolerated and safe to use. The intervention with TXA mouthwash is also affordable, readily available on the market, and therefore easy to implement in clinical practice.

In our study, 6 patients in the TXA group and 9 patients in the placebo group interrupted their NOAC (without it being planned in preparation for surgery). Unplanned interruption of anticoagulants is a concern in clinical practice, since it is associated with increased risk of cardiovascular events [13]. Patient adherence and compliance with the doctor’s advice remain important.

An exploratory subgroup analysis showed that patients who underwent extraction of 2 or more teeth and elderly patients and patients who restarted their NOAC within 24 hours after the dental extraction might benefit more from treatment with TXA. However, the latter finding was not statistically significant. These findings support the hypothesis that the potential benefit of a hemostatic agent is likely to be the largest in patients with friable mucosa and more extensive tissue damage. An immature coagulum combined with reinitiation of the anticoagulant may then result in bleeding. Future studies may focus on this high-risk population.

After enrolling 222 out of 236 planned patients, the trial was prematurely stopped during the SARS-CoV-2 pandemic after a futility analysis by the Data Safety Monitoring Board based on the number of patients with oral bleeding. This was the main limitation of the study. Another limitation was that we relied on self-reported information gathered during a follow-up by phone to assess the patients’ compliance with the study protocol. However, the studied intervention was a simple intervention, and patients got both oral and written instructions on a schematic leaflet about the protocol. A last limitation was that there was no fixed time point on restarting the NOAC after dental extraction. Patients were instructed on when to restart their NOAC, but the exact timing of the NOAC re-intake was left at the patient’s decision. As a result, there was a spread in the timing of the NOAC restart among the studied patients, and the possible effect hereof on bleeding after dental extraction is unclear. The question on timing of NOAC restart needs further study.

In conclusion, a 10% TXA mouthwash did not reduce the number of patients with any oral bleeding after dental extraction, nor the periprocedural bleeding or early bleeding after extraction. TXA appears to reduce delayed bleeding and bleeding after multiple dental extractions. Follow-up studies may explore potential benefit in high-risk patient populations.

## Supporting information

S1 CONSORT Checklist The current manuscript follows the CONSORT reporting guidelines. CONSORT, Consolidated Standards of Reporting Trials.
(PDF)

S1 SAP Interim The interim statistical analysis plan of the EXTRACT-NOAC study. NOAC, non-vitamin K oral anticoagulant.
(PDF)

S2 SAP Final The final statistical analysis plan of the EXTRACT-NOAC study. NOAC, non-vitamin K oral anticoagulant.
(PDF)

S1 Table Details of the dental extraction are given according to the treatment groups. (PDF)

S2 Table Details of the unplanned medical contacts and unplanned NOAC interruptions are given according to the treatment groups. NOAC, non-vitamin K oral anticoagulant.
(PDF)

## Abbreviations

CI: confidence interval
CONSORT: Consolidated Standards of Reporting Trials
DOAC: direct K oral anticoagulant
EHRA: European Hearth Rhythm Association
NOAC: non-vitamin K oral anticoagulant
SARS-CoV-2: Severe Acute Respiratory Syndrome Coronavirus 2
TXA: tranexamic acid
