# Supplementary material for: Tranexamic acid and bleeding in patients treated with non-vitamin K oral anticoagulants undergoing dental extraction: The EXTRACT-NOAC randomized clinical trial
Source: PLoS Med. 2021 May 3;18(5):e1003601. doi: 10.1371/journal.pmed.1003601 (PMC8128271; doi:10.1371/journal.pmed.1003601)
Supplement: S1 SAP Interim — NOAC, non-vitamin K oral anticoagulant. (PDF) [file pmed.1003601.s002.pdf]

# EXTRACT-NOAC: Interim Statistical Analysis Plan

Date: 09 June2020

Version Final 1.0

Author: Ann Belmans, Study Statistician

## 1. Signatures

Principal Investigator: Prof. Dr. Peter Verhamme

Signed: ______________________ Date: 9 / JUN / 2020

Trial Statistician: Ann Belmans, M. Sc.

Signed: ______________________ Date: 9 Jun 2020

DSMB Chairman: Prof. Dr. K. Peerlinck

Signed: ______________________ Date: 8/6/'20

## 2. Table of Contents

EXTRACT-NOAC: Interim Statistical Analysis Plan ..... 1
1. Signatures ..... 1
2. Table of Contents ..... 2
3. Background ..... 2
4. Purpose ..... 2
5. Software ..... 2
6. Sample Size ..... 2
7. Futility Assessment & Re-Estimation of Sample Size ..... 3
8. References ..... 4
Appendix I ..... 5

## 3. Background

The EXTRACT-NOAC trial assesses whether treatment with tranexamic acid can reduce bleeding in patients treated with oral anticoagulants undergoing dental extraction.

The study was designed as a fixed sample size study, whereby a total of 236 patients (118 per group) was to be included in the study.

In March 2020, at the time of the outbreak of the covid-19 pandemic, the trial was suspended. At that time, a total of 222 patients had been randomised into the study of which 218 were evaluable.

In May 2020, it was decided that clinical studies could restart in June 2020 under strict circumstances that limit the spread of COVID-19 between study subjects and investigators.

Regarding the EXTRACT-NOAC study, it was decided to perform a futility analysis prior to restarting. If the chance of obtaining a positive study result is too low, the study will not be re-initiated. At the same time as the futility analysis, a sample size re-evaluation will be done.

All calculations and evaluations of the primary efficacy endpoint regarding the futility and sample size reevaluation are to be done by the independent DSMB and DSMB statistician, i.e. the study team will remain blinded to all study results.

## 4. Purpose

The purpose of this document is to describe the statistical methodology that is to be used by the DSMB statistician for the assessment of futility and reevaluation of the sample size.

## 5. Software

All evaluations will be done using East 6.5.

## 6. Sample Size

The primary outcome is post-extraction bleeding. It was assumed that 30% of patients in the control group would experience a bleeding event and that this risk could be reduced by 15% (absolute), i.e. that the risk of bleeds was 15% in the intervention group.

Therefore, 118 patients per group were to be recruited in order to achieve 80% power to detect a statistically significant effect at a 2-sided significance level of 5%.

## 7. Futility Assessment & Re-Estimation of Sample Size

The re-evaluation of the sample size will be done using the methods described by Chen, DeMets en Lan (2004).

The conditional power at the original sample size of 236 patients, $CP_{\widetilde{\delta_1}}$,will be calculated based on the event rates estimated from the interim data.

The range of conditional powers will be divided into 3 zones:

a) Unfavorable: $CP_{\widetilde{\delta_1}} < CP_{min}$: when the conditional power falls within this zone, the study will not be reinitiated.
b) Promising: $CP_{min} \leq CP_{\widetilde{\delta_1}} < 1 - \beta$: when the conditional power falls within this zone, the sample size will be extended to a maximum of 300 patients in total.
c) Favorable: $CP_{\widetilde{\delta_1}} \geq 1\text{-}\beta$: when the conditional power is above the initial target of 80%, the trial will proceed to the originally planned sample size of 236 patients.

The target power for the final analysis will be 80%.

The choice of $CP_{min}$ affects the chance of the study being stopped at this stage and the power that will be achieved for the final analysis. Therefore, simulations were performed to assess the effect of the choice of $CP_{min}$ on the chance of the study being stopped and the power of the study.

Simulations were done under the assumptions made for the initial sample size calculations and the above proposed method of stopping or extending the study and for a range of smaller 'true' effect sizes:

1. Power 80%
2. 2-sided significance level of 5%
3. Binary outcome
4. Event rate in Control group of 30%
5. Event rates in interventional group of 15%, 16%, 17%, 18%, 19% or 20%.
6. Sample size reassessment when 94% (222/236) of data available, i.e., information fraction of 94%
7. Extension of total sample size possible with a factor of 1.274 (=300/236).
8. No stopping for early efficacy
9. Above method for terminating the study, extending the study or finishing the study as planned whereby $CP_{min}$ is assumed to be 30%, 50% or 60%.

For each scenario, a total of 10,000 simulations were done. The results can be found in Appendix 1.

For $CP_{min}$=50, the figure below illustrates the sample size extension across the possible values for $CP_{\widetilde{\delta_1}}$ (0 to 1).

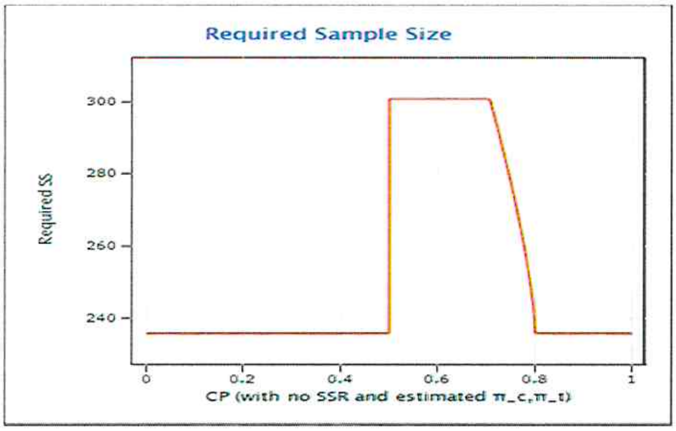


Based on the results of these simulations, $CP_{min}$ will be set at 50%.

By only extending the sample size when the conditional power is in the favorable zone above 50%, it will be appropriate to use the classic Wald test for statistical analysis without increasing the Type I error.

## 8. References

Chen JYH, DeMets DL, Lan KKG.(2004). Increasing the sample size when the unblinded interim result is promising. *Statistics in Medicine*, 23(7), 1023-1038.

## Appendix I

| | | Fixed Sample Size | Sample Size Reassessment | | | | | | | | |
|---|---|---|---|---|---|---|---|---|---|---|---|
| | | | | Unfavorable, i.e. study stopped | | Promising, i.e. study extended | | Favorable, i.e. study as planned | | Promising or Favorable | |
| $CP_{min}$ | Event rate intervention | Power | Overall Power | % of simulations | Power$ | % of simulations | Power | % of simulations | Power | % of simulations | Power |
| 30% | 20% | 42.5% | 45.8% | 49.0% | 5.5% | 18.0% | 65.9% | 33.0% | 94.6% | 51.0% | 84.4% |
| | 19% | 50.9% | 53.0% | 42.2% | 7.1% | 17.8% | 68.4% | 40.0% | 94.8% | 57.8% | 86.7% |
| | 18% | 58.1% | 60.2% | 36.0% | 8.9% | 17.6% | 71.0% | 46.4% | 96.0% | 64.0% | 88.5% |
| | 17% | 65.8% | 69.4% | 28.0% | 10.7% | 17.2% | 76.7% | 54.8% | 97.0% | 72.0% | 92.9% |
| | 16% | 73.4% | 76.1% | 22.2% | 12.0% | 15.9% | 81.9% | 61.9% | 97.5% | 77.8% | 94.7% |
| | 15% | 80.0% | 81.7% | 17.3% | 14.9% | 13.6% | 83.0% | 69.1% | 98.0% | 82.7% | 95.7% |
| 50% | 20% | 42.5% | 43.8% | 56.5% | 9.7% | 11.0% | 70.0% | 32.5% | 94.4% | 43.5% | 88.3% |
| | 19% | 50.9% | 49.1% | 50.1% | 11.7% | 11.3% | 71.4% | 38.6% | 94.7% | 49.9% | 86.7% |
| | 18% | 58.1% | 58.5% | 43.4% | 14.5% | 10.9% | 79.3% | 45.7% | 96.4% | 56.6% | 92.9% |
| | 17% | 65.8% | 67.7% | 35.0% | 17.5% | 10.7% | 82.8% | 54.3% | 97.0% | 65.0% | 94.7% |
| | 16% | 73.4% | 74.7% | 27.7% | 20.2% | 10.2% | 83.9% | 62.1% | 97.9% | 72.3% | 95.5% |
| | 15% | 80.0% | 81.3% | 21.5% | 24.0% | 9.4% | 87.8% | 69.1% | 98.3% | 78.5% | 96.2% |
| 60% | 20% | 42.5% | 42.5% | 60.6% | 12.0% | 7.4% | 69.2% | 31.9% | 93.8% | 39.4% | 89.2% |
| | 19% | 50.9% | 49.6% | 53.4% | 14.5% | 7.5% | 72.1% | 39.1% | 93.8% | 46.6% | 90.3% |
| | 18% | 58.1% | 58.5% | 46.6% | 18.2% | 7.6% | 77.7% | 46.0% | 96.7% | 56.3% | 94.0% |
| | 17% | 65.8% | 66.2% | 38.8% | 22.4% | 7.4% | 80.4% | 53.8% | 97.0% | 61.2% | 95.0% |
| | 16% | 73.4% | 73.2% | 32.7% | 25.1% | 6.2% | 84.4% | 61.1% | 97.7% | 67.3% | 96.5% |
| | 15% | 80.0% | 80.0% | 25.7% | 30.9% | 5.8% | 89.4% | 68.5% | 98.3% | 74.3% | 97.6% |

$ Percentage of unfavorable studies that would have yielded a significant result if they had carried on as planned to 236 patients.
The event rate of the control group is assumed to be 30%.
