# Supplementary material for: Tranexamic acid and bleeding in patients treated with non-vitamin K oral anticoagulants undergoing dental extraction: The EXTRACT-NOAC randomized clinical trial
Source: PLoS Med. 2021 May 3;18(5):e1003601. doi: 10.1371/journal.pmed.1003601 (PMC8128271; doi:10.1371/journal.pmed.1003601)
Supplement: S2 SAP Final — NOAC, non-vitamin K oral anticoagulant. (PDF) [file pmed.1003601.s003.pdf]

EXTRACT-NOAC Study

EXTRACT-NOAC Specific Statistical Analysis Plan

Final Version 1.0

Project Title: Tranexamic acid to reduce bleeding in patients treated with non-vitamin k oral anticoagulants undergoing dental extraction: the EXTRACT-NOAC trial

Randomized, double-blind, placebo-controlled, multi-center phase IV clinical trial assessing the benefit of TXA mouthwash.

Date: 18/03/2021

1 Contents

1 Contents 2
2 Purpose 3
3 Description of the Study 3
4 Master Statistical Analysis Plan 3
5 Study Objectives and Endpoints 3
5.1 Study Objective 3
5.2 Study Outcomes 4
5.2.1 Primary Outcome 4
5.2.2 Secondary Outcomes 4
5.2.3 Safety outcomes 4
5.2.4 Exploratory outcomes 5
6 Study Methods 5
6.1 Overall Study Design and Plan 5
6.2 Selection of Study Population 5
6.2.1 Participant Inclusion Criteria 5
6.2.2 Participant Exclusion Criteria 5
6.2.3 Strategies for recruitment and retention 5
7 Sequence of Planned Analyses 6
7.1 Interim Analyses 6
7.1.1 Additional Sub-group Analyses 6
7.1.2 Multiple Comparison/Multiplicity 6
7.1.3 Tabulation of Individual Response Data 6
7.1.4 Exploratory Analyses 6
8 Final Analysis and Reporting 6
9 Sample Size Determination 6
11. Analysis Populations 7
9.1 Full Analysis Set (FAS) 7
9.2 Safety Set (SS) 7
9.3 Per Protocol Set (PPS) 7
12. General Issues for Statistical Analysis 7
12.1 Analysis Software 7
12.4 Analysis of the Primary Efficacy Outcome(s) 8
13 Methods for Missing Data 8
14 Multiple Comparisons 8
15 References 9

2 Purpose

In addition to the Global SAP, this document provides further details for the statistical evaluation of the primary, secondary endpoints of the EXTRACT-NOAC study.

3 Description of the Study

This is a randomized, double-blind, placebo-controlled, multi-center phase IV clinical trial assessing the benefit of TXA mouthwash with a total of 236 randomized patients.

4 Master Statistical Analysis Plan

This EXTRACT-NOAC -Specific SAP provides additional and specific details about the statistical methods for the evaluation of the outcomes.

5 Study Objectives and Endpoints

5.1 Study Objective

The primary objective of EXTRACT-NOAC study is to evaluate whether TXA mouthwash reduces oral bleeding in patients who undergo dental extraction and are treated with NOACs.

Secondary objectives:

- To evaluate whether TXA mouthwash before the extraction reduces the bleeding during the procedure.
- To evaluate whether TXA mouthwash reduces early and/or delayed bleeding events
- To evaluate whether TXA mouthwash reduces minor, clinically relevant or major bleeding events or reinterventions.
- To evaluate whether TXA mouthwash reduces the number of unplanned NOAC interruptions.
- To evaluate the safety of skipping the dose of the NOAC on the day of the extraction.

Exploratory analyses:

- To investigate the potential relationship between time of last NOAC dose administered prior to the extraction (regardless of specific NOAC type), time of resuming NOAC after the procedure, and bleeding events
- To investigate the potential benefit of TXA in subgroups of patients (molar versus non-molar extraction)

5.2 Study Outcomes

The procedural bleeding score is a visual analogue score (VAS) ranging from 0 (no bleeding) to 10 (unstoppable bleeding). A reintervention is defined as any procedure in the oral cavity for the treatment of bleeding, performed by any dentist or maxillofacial surgeon, except for rinsing the extraction socket with saline. All suspected bleeding events and reinterventions will be adjudicated by a blinded adjudicator, unaware of the assigned treatment. Bleedings are defined as the following:

- Minor bleeding: any oral bleeding experienced by the patient that does not require medical contact. Eg. blood on the pillow, bleeding requiring the use of additional gauzes, clear red bleeding when spitting out the mouthwash
- Clinically relevant non-major bleeding: any oral non-major bleeding requiring unplanned medical contact (by phone or with any health care professional (dentist, general practitioner, maxillofacial surgeon), with or without re-intervention
- Major bleeding: any oral bleeding requiring blood transfusion, hospitalization or resulting in death
- Early bleeding: any oral bleeding occurring after the extraction up to and including day 1 after the extraction
- Delayed bleeding: any oral bleeding occurring between day 2 and day 7 after the dental extraction

5.2.1 Primary Outcome

Any oral bleeding (early or delayed; minor or clinically relevant).

5.2.2 Secondary Outcomes

- The procedural bleeding score
- Early and delayed bleeding
- Minor, clinically relevant non-major and major bleeding
- Clinically-relevant bleeding (non-major and major)
- The number of reinterventions
- The number of unplanned NOAC interruptions

5.2.3 Safety outcomes

- Any non-oral bleeding
- All thrombotic events including myocardial infarction, stroke, systemic embolism and venous thrombo-embolism up to the endo of study follow-up.
- Any allergic reaction

5.2.4 Exploratory outcomes

The following subgroup analyses are planned:

- Risk factor analysis for bleeding, eg. patient demographics and procedural characteristics
- The number of bleeding events in patients in relation to the time since stopping and resuming NOAC in relation to the dental extraction, e.g. in patients who stopped/resumed NOAC treatment within 24 hours prior/after extraction compared to more than 24 hours prior/after extraction.

6 Study Methods

6.1 Overall Study Design and Plan

Patients will be randomized through an interactive web recognition system to a 1g/10mL (10%) TXA mouthwash or a taste and color-matching placebo, both manufactured by the Leuven Centre for Clinical Pharmacology, University Hospitals Leuven, Belgium. Patients will be enrolled in the study and assigned to the study interventions by clinicians. (Patients, clinicians and treating surgeons will be blinded to the allocated intervention.)

6.2 Selection of Study Population

6.2.1 Participant Inclusion Criteria

- Patients scheduled for dental extraction and treated with edoxaban, apixaban, rivaroxaban or dabigatran
- Not having taken the NOAC on the day of the extraction
- Provision of signed and dated informed consent form
- Stated willingness to comply with all study procedures and availability for the duration of the study
- 18 years or older

6.2.2 Participant Exclusion Criteria

- Subjects with any condition that as judged by the Investigator would place the subject at increased risk of harm if he/she participated in the study.
- Pregnancy or lactation
- Known allergic reaction to TXA

6.2.3 Strategies for recruitment and retention

All patients undergoing dental extraction at the outpatient clinic of the registered sites will be screened for eligibility before their dental extraction. Standard-of-care (discontinuation of the NOAC on the day of the procedure) is already implemented in the participating site. Adherence to the study protocol will be assessed by phone call on day 2 and day 7

7 Sequence of Planned Analyses

7.1 Interim Analyses

After inclusion of 100 patients, a safety reviewed is planned to assess thrombotic events, allergic reaction and non-oral bleeding. After 100 patients have reached their 7-day visit, an efficacy review is planned to reassess the required sample size. This will be done using the methods described by Cui, Hung & Wang (1999). The sample size re-estimation (SSR) will be based on the observed conditional power at the time of the interim analysis. The re-estimated sample size will be required to be between 150 and 300 patients. Full details for the methodology of the SSR is provided in an Interim Statistical Analysis Plan.

7.1.1 Additional Sub-group Analyses

A number of exploratory subgroup analyses is planned, for which the study is not powered, including:

The number of bleeding events in patients who resumed their NOAC treatment within 24 hours after extraction compared to later than 24 hours after extraction, stratified per treatment group.

Risk factor analysis for bleeding, eg. patient demographics and procedural characteristics

7.1.2 Multiple Comparison/Multiplicity

A limited number of predefined subgroup analyses will be tested. No adjustment to the significance level will be made.

7.1.3 Tabulation of Individual Response Data

Individual data will be collected and stored.

7.1.4 Exploratory Analyses

Defined under subgroup analysis.

8 Final Analysis and Reporting

Upon final database lock, statistical analyses of the data will be performed according to the methods described in this document and the Master SAP.

9 Sample Size Determination

Based on the previously cited pilot study, we estimate the rate of the primary outcome at 30% in the placebo group. We estimate to show an absolute risk reduction (ARR) of 15% This means an

incidence of 15% in the tranexamic acid group. Confidence level is set to 95%, power is set to 80%. Under the above assumptions, in order to have 80% to detect a statistically significant risk difference between treatments, 118 patients per group or 236 patients in total will be required.

11. Analysis Populations

The following analysis sets will be of interest:

9.1 Full Analysis Set (FAS)

The full analysis set will be included all randomized patients, and excluded patients for whom no follow-up data could be obtained or control patients who used off-trial prophylactic TXA [4].

9.2 Safety Set (SS)

No separate Safety Set will be defined.

The FAS will be used for the evaluation of all safety parameters.

9.3 Per Protocol Set (PPS)

If warranted, patients from the FAS with major protocol deviations will be excluded from the per protocol set (PPS). The PPS will be used for the evaluation of all efficacy endpoints.

12. General Issues for Statistical Analysis

12.1 Analysis Software

All analyses will be performed using SAS version 9.4 [3] and SAS/STAT version 15.1 for Windows.

12.2 Summary Statistics

Continuous variables will be presented by randomized treatment group by mean, standard deviation, median and interquartile range, categorical variables will be summarized by observed frequencies and proportions. The comparisons between groups for continuous variables will be performed by using t-test (if normally distributed), or by using Wilcoxon rank-sum test (if the data shows deviations from normal distribution). Chi-square or Fisher's exact test (if cells with expected counts of <5 patients) will be used for the comparisons of categorical variables between groups.

12.3 General Issues

The overall significance level for the primary endpoint of this study is set at 5% (2-sided). To account for the planned interim analysis, the methods as described by Cui, Hung & Wang (1999) will be used to preserve the overall Type I error. For the secondary endpoints, no adjustment will be made to the significance level to account for multiple testing.

For all primary and secondary endpoints, an estimate of the treatment effect and associated 95% confidence interval will be presented. E.g. for the primary endpoint, the risk difference and its 95% confidence interval will be presented.
Due to the randomized and double-blind nature of the study, no significance testing of baseline characteristics will be done.

12.4 Analysis of the Primary Efficacy Outcome(s)

The number of bleeding events up to day 7 will be analyzed in terms of a risk difference between the two treatment groups, using the methods described by Cui, Hung & Want (1999). The primary outcome (i.e. number of patients with any oral bleeding) will be analyzed using a chi-square test. The treatment effect will be estimated by the risk ratio. The risk ratio and its corresponding 95% confidence intervals (CI) will be presented. This analysis will be based on the ITT population. Same analysis based on PP population will also be performed as a sensitivity analysis.

12.5 Analysis of the Secondary Outcome(s) and Sub-group Analysis

The secondary outcomes with a pre-specified subgroup analysis will be performed for number of bleeds and the number of patients with bleeds.

The number of the bleeds will be analyzed by using negative binomial regression model by using PROC GENMOD (SAS procedure) [2]. The length of follow-up will be considered as an offset variable. For the sub-group analysis, the model will include factor for randomized treatment, the sub-group and their interaction effect. The estimated treatment effect will be presented as rate ratio, with corresponding 95% confidence interval (CI), and two-sided p-value.

The number of patients with bleeds outcomes will be analyzed by means of a logistic regression. For the sub-group analysis, the logistic regression model will include factor for randomized treatment, the sub-group and their interaction term.

13 Methods for Missing Data

Not applicable.

14 Multiple Comparisons

Since only 1 primary endpoint is defined, no adjustment of the significance level is required.

For secondary efficacy endpoints, due to the exploratory nature of the efficacy analyses, no adjustment for multiple comparisons will be made.

15 References

1. Cui, L., Hung, H.M.J. and Wang, S.-J. (1999), Modification of Sample Size in Group Sequential Clinical Trials. Biometrics, 55: 853-857
2. Gardner, W., Mulvey, Edward P., Shaw, Esther C., 1995. Regression Analysis of Counts and Rates: Poisson, Overdispersed Poisson, and Negative Binomial Models. Psychological Bulletin, Vol. 118, No 3 392-404
3. SAS software, version 9.4 of the SAS System for Windows. Copyright © 2002 SAS Institute Inc. SAS and all other SAS Institute Inc. product or service names are registered trademarks or trademarks of SAS Institute Inc., Cary, NC, USA
4. The European Medicines Agency's (EMEA's) (2006) Guidelines on Statistical Principles for Clinical Trials: ICH Topic E9. Eur Med Agency.
