# Supplementary material for: Tranexamic acid and bleeding in patients treated with non-vitamin K oral anticoagulants undergoing dental extraction: The EXTRACT-NOAC randomized clinical trial
Source: PLoS Med. 2021 May 3;18(5):e1003601. doi: 10.1371/journal.pmed.1003601 (PMC8128271; doi:10.1371/journal.pmed.1003601)
Supplement: S1 Table — (PDF) [file pmed.1003601.s004.pdf]

**S1 Table. Details of dental extractions according to treatment group.**		
**Variable**	**Tranexamic acid (N=106)**	**Placebo (N=112)**
**Indication of dental extraction – no. (%)***	-	-
Abscess	23 (21.7)	20 (17.9)
Periodontal disease	37 (34.9)	33 (29.5)
Tooth decay	70 (66.0)	74 (66.1)
Other indications†	5 (4.7)	10 (8.9)
**Surgical procedures – no. (%)***	-	-
Stitches	105 (99.1)	111 (99.1)
Burring	23 (21.7)	28 (25.0)
Additional hemostatics‡	48 (45.3)	39 (34.8)
Antibiotics	22 (20.8)	27 (24.1)
Chlorhexidine mouthwash prescribed	48 (45.3)	66 (58.9)
Leukocyte- and Platelet-Rich Fibrin	9 (8.6)	17 (15.2)
**Number of extracted teeth – no. (%)**	-	-
1 tooth	45 (42.5)	55 (49.1)
2 teeth	14 (13.2)	18 (16.1)
3-4 teeth	22 (20.8)	21 (18.8)
≥ 5 teeth	25 (23.6)	18 (16.1)
**Maxilla or mandible – no. (%)**	-	-
Maxilla	40 (37.8)	41 (36.6)
Mandible	43 (40.6)	48 (42.9)
Maxilla and mandible	23 (21.7)	23 (20.5)
**Incisors/canines or molars – no. (%)**	-	-
Incisors/canines	16 (15.1)	12 (10.7)
Molars	54 (50.9)	70 (62.5)
Incisors or canines and molars	36 (34.0)	30 (26.8)

Number of patients are reported. Percentages may not total 100 because of rounding.
*These data are not mutually exclusive.
†Other most-common indications for tooth extraction were root resorption, tooth extraction prior to organ transplantation or to an oncological treatment or to the use of bisphosphonates.
‡Additional hemostatics included gauze compression and resorbable oxidized cellulose materials.
