# Supplementary material for: Tranexamic acid and bleeding in patients treated with non-vitamin K oral anticoagulants undergoing dental extraction: The EXTRACT-NOAC randomized clinical trial
Source: PLoS Med. 2021 May 3;18(5):e1003601. doi: 10.1371/journal.pmed.1003601 (PMC8128271; doi:10.1371/journal.pmed.1003601)
Supplement: S2 Table — NOAC, non-vitamin K oral anticoagulant. (PDF) [file pmed.1003601.s005.pdf]

S2 Table. Details of unplanned medical contact and NOAC interruption.		
**Outcome**	**Tranexamic acid (N=106)**	**Placebo (N=112)**
**Unplanned medical contact – no. (%)***	-	-
Bleeding	7 (6.6)	15 (13.4)
Infection	3 (2.8)	7 (6.3)
Other	0	5 (4.5)
**NOAC interruption – no. (%)**	-	-
Bleeding	3 (2.8)	6 (5.4)
Accidental	1 (0.9)	2 (1.8)
Doctor’s advice	1 (0.9)	0
Patient’s preference	1 (0.9)	1 (0.9)
In preparation for surgery	2 (1.9)	3 (2.7)

Number of patients are reported.

*These data are not mutually exclusive.
